# Improving Safety through a Virtual Learning Collaborative

**DOI:** 10.1097/pq9.0000000000000740

**Published:** 2024-07-19

**Authors:** Jeffrey P. Durney, Katie M. Catalano, D. Marlowe Miller, Amy J. Starmer, Kate Humphrey, Catherine Perron, Anne M. Stack

**Affiliations:** From the * Department of Pediatrics, Boston Children’s Hospital, Boston, Mass.; † Program for Patient Safety and Quality, Boston Children’s Hospital, Boston, Mass.; ‡ Division of Emergency Medicine, Department of Pediatrics, Boston Children’s Hospital, Boston, Mass.

## Abstract

**Background:**

Frontline healthcare safety leaders require expertise and confidence to manage local safety programs effectively yet are confronted with substantial challenges in identifying risk and reducing harm.

**Methods:**

We convened a multidisciplinary safety learning collaborative in a children’s hospital pediatric department and used the Institute for Healthcare Improvement’s Breakthrough Series model. Participants attended four virtual education sessions over 13 months (September 2020–September 2021) focused on identifying harm and using tools to improve safety. We analyzed departmental safety data monthly throughout the collaborative. The primary outcome was the development of improvement projects using direct application of the session content. The secondary outcome was participant confidence in improving safety via pre- and postsurveys.

**Results:**

Seventy clinicians and quality consultants participated. Fifteen divisional safety improvement projects were initiated. The percentage of survey respondents who reported feeling “completely confident” in their ability to improve safety increased from 26% (n = 39) to 58% (n = 26) from September 2020 to September 2021 (*P* = 0.01) and maintained at 65% 1 year after the end of the collaborative. We observed a decrease in the mean rate of reported inpatient preventable and possibly preventable moderate/serious/catastrophic events per 1000 bedded days from 1.10 (baseline) to 0.71 (intervention period).

**Conclusions:**

Through a collaborative effort in a virtual learning environment, we facilitated the development of fifteen safety projects, increased leaders’ confidence in improving safety, and saw improved inpatient safety. This approach, which involves healthcare professionals from various disciplines, may be effectively adapted to other settings.

## INTRODUCTION

Healthcare professionals are challenged to identify sources of risk and develop effective interventions to protect patients from harm. Ever since The Institute of Medicine report *To Err Is Human: Building a Safer Health System* was published in 2000, a shift has occurred regarding human error and the degree to which some types of harm may be avoidable.^1^ A 2015 report by an expert panel convened by The National Patient Safety Foundation suggests that patient safety remains a thorny public health issue despite some progress. As a result, the expert panel put forth targeted recommendations with action steps, including: (1) ensuring that leaders establish and sustain a safety culture, (2) creating centralized and coordinated oversight of patient safety, (3) addressing safety across the entire care continuum, and (4) supporting the health care workforce.^2^ Much work remains to ensure patients are free from preventable harm. This goal is attainable and essential by taking a system-level approach.^3^

In 2020, our hospital leadership distributed a safety culture survey, a subset of the Agency for Healthcare Research and Quality Hospital survey (version 2.0),^4^ to all faculty and staff. At the same time, results demonstrated improved scores in many domains from the prior survey in 2018; performance in the overall perception of patient safety left room for further improvement. The need for education among frontline safety leaders to systematically manage threats in a busy clinical environment was evident.

Coupling the recommendation from the National Patient Safety Foundation expert panel with the results from our internal safety culture survey, the Department of Pediatrics Quality Program leaders aimed to plan and execute a multidisciplinary learning collaborative to support local leaders in managing their safety programs. Our primary goal was to improve safety and reduce potential harm by initiating local safety improvement projects.

## METHODS

### Context

The intervention occurred within a department of pediatrics in a free-standing academic children’s hospital with approximately 400 beds. There are 15 clinical divisions and over 800 departmental faculty. Each division uses a triad model consisting of a physician, nurse, and quality improvement consultant to manage quality and safety locally. (**See figure, Supplemental Digital Content 1, which displays Department of Pediatrics Quality Improvement Work Structure.** http://links.lww.com/PQ9/A565.) A centralized team at the departmental level, consisting of a director, program manager, 3 quality improvement consultants and 2 data analysts, supports quality and safety management. In turn, these groups work within a hospital-level quality and safety program.

**Fig. 1.**
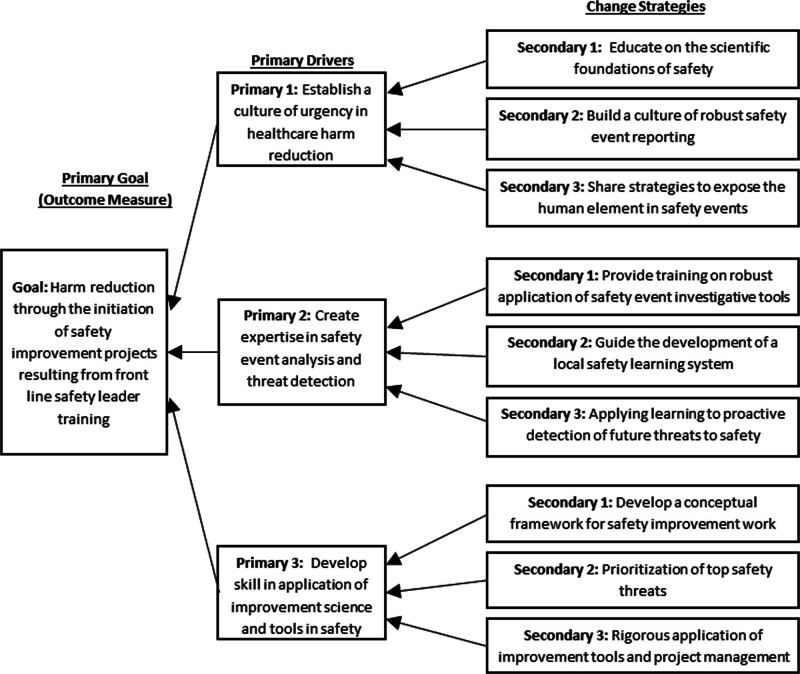
Improving departmental safety key driver diagram: The development of the key driver diagram allowed us to explore the drivers and potential change strategies to determine the content of our learning session.

Initially, we established a multidisciplinary steering committee composed of safety and quality leaders across the organization. The committee developed a key driver diagram to organize the work and determine key drivers and change strategies to achieve our aim (Fig. 1). The committee also planned the design and execution of the collaborative. We utilized the Institute for Healthcare Improvement’s Breakthrough Series (BTS) model. This approach consists of a short-term learning engagement convening representatives from several organizations or individual areas. The goal is to impart knowledge to be directly applied to address challenges at the local level.^5^ Session participants included representatives from all clinical divisions in the department. Disciplines of participants included physicians, nurses, and quality improvement consultants. In some cases, divisional quality and safety leaders had not received formal training in the science of safety and expressed the desire for more education to improve their safety programs.

### Intervention(s)

Our primary aim was to increase the ability of leaders to improve safety and reduce potential harm in their local areas through the initiation of safety improvement projects. Implementation was done on a local level using quality improvement consultants as facilitators. A secondary aim was to increase clinician confidence in improving safety in their division. We aimed to improve the rate of reported near misses, none or minor preventable and possibly preventable events, a signal for threat detection, and decrease the reported serious and possibly preventable events. At our institution, safety events are classified by local reporters and are reviewed, sometimes amended, and finalized by the hospital safety team. Our classification system indicates the severity and preventability of the event (**See table, Supplemental Digital Content 2,** which shows event classification levels. http://links.lww.com/PQ9/A566).

#### Learning Sessions

**Fig. 2.**
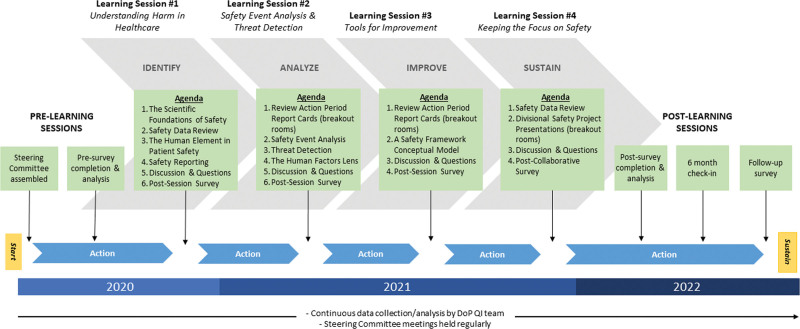
Learning collaborative timeline and learning session details: The timeline and learning session agendas supported learning collaborative planning and implementation. DoP, Department of Pediatrics; QI, quality improvement.

We designed and facilitated four 2-hour sessions over 1 year focused on specific areas of local safety management, including (1) understanding harm in healthcare, (2) safety event analysis and threat detection, (3) tools for improvement, and (4) keeping the focus on safety (Fig. 2). Selected faculty members with expertise in safety and quality led the sessions. Although the intent was to meet in person, challenges associated with the COVID-19 pandemic necessitated virtual sessions facilitated by a remote meeting platform. We selected a videoconferencing platform (Zoom by Zoom Video Communications, San Jose, Calif., zoom.us) to deliver all didactic content and facilitate communication with participants. Virtual education in the face of a global pandemic has been demonstrated to be effective in maintaining progress in quality improvement education efforts without exposing staff to the risk of in-person gatherings.^6^

Participants completed action-based report cards to reinforce learning between sessions. The teams presented their safety projects to the participants during the final learning session. We also coordinated the American Board of Pediatrics Maintenance of Certification Part 4 credit to strengthen physician engagement.

#### Operational Framework

**Fig. 3.**
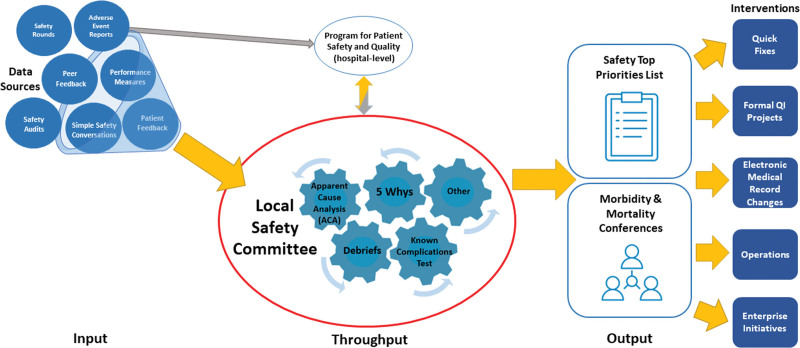
Safety management framework: The development and application of the safety management framework provided a systematic approach to capturing, analyzing, and mitigating threats and events at a local level.

We developed and shared an operational framework (Fig. 3) to aid participants in local safety management. This framework focuses on three areas within local safety management: Input: Harnessing a broad range of threats and event reports; Throughput: Using validated tools (eg, apparent cause analysis, 5-whys, event debriefs, known complications tests, etc.) to analyze events and improve safety; and Output: Organizing suggested action steps for managing safety threats and events.

#### Surveys

Before beginning the training program, we administered a survey to identify baseline safety management practices and assess participant confidence to identify the focus of learning sessions. Using the Kirkpatrick Model of Training Evaluation, we leveraged four immediate postsession surveys to assess learning and collect feedback to enhance future sessions. This model provides a comprehensive understanding of training effectiveness by assessing participant reactions, measuring learning outcomes regarding knowledge and skills, evaluating behavior or performance, and analyzing overall results.^7^ Throughout the learning sessions, we invited divisional leaders to share progress and barriers with peers through facilitated group discussions. We utilized existing departmental quality forums for project updates to reinforce adoption and facilitate learning. Moreover, we presented opportunities in established divisional quality meetings to monitor progress and provide support. A postsurvey was administered immediately after the final learning session and 1-year following completion to measure the durability of the training.

## STUDY OF THE INTERVENTIONS

For the primary outcome, we aimed to facilitate the development of at least 1 safety improvement project per division. We sought to integrate learned safety principles to lead to harm reduction due to collaboration. For the secondary aims, we analyzed pre- and postintervention survey data for participant confidence in improving safety locally: preintervention (September 2020), immediate postintervention (September 2021), and 1 year later (September 2022) (**See table, Supplemental Digital Content 3,** which shows pre and postcollaborative survey questions. http://links.lww.com/PQ9/A567). We collected and managed survey data using REDCap electronic data capture tools hosted at Boston Children’s Hospital.^8,9^ We also analyzed departmental safety data over time.

## MEASURES

The primary outcome measure was the number of safety projects launched by participating safety leaders. For the secondary outcomes, we measured the percent change in respondents’ confidence level in their ability to improve safety locally as reported on the three surveys from September 2020 to September 2022. The survey question was, “I feel confident in my ability to improve safety within my division.” The question had the following anchored responses: completely confident, fairly confident, somewhat confident, slightly confident, and not confident. Another secondary outcome measure was the rate of reported near misses, none, and minor and possibly preventable events, as well as the reported moderate, serious, and catastrophic preventable and possibly preventable events found in department-wide safety data. The process measure was the proportion of participants who attended at least three learning sessions.

## ANALYSIS

We calculated the number of local safety projects initiated. To assess provider confidence in improving safety, we compared scores for the question “I felt confident in my ability to improve safety within my division,” using a 5-point Likert scale and dichotomized the most positive response (completely confident) versus all other response types on the three pre- and postsurveys. We used Fisher exact test to determine if the percent change in survey scores was statistically significant.

We tracked departmental safety data by location (emergency, inpatient and ambulatory). We created u-charts or g-charts, depending on the total number of events, using SQC Pack version 7.0 (PQ Systems, Dayton, Ohio). We established an initial baseline period before the start of the COVID-19 pandemic (March 2020) and a new baseline for the period leading up to the launch of the collaborative. We applied standard control chart rules to indicate special cause variation during the period following the launch of the collaborative. For the u-charts, we normalized the data to 1000 patient visits/inpatient days over 42 months.^10^ For the g-charts, we measured the days between events. We calculated the percentage of participants who enrolled in the collaborative and attended at least three sessions.

## ETHICAL CONSIDERATIONS

This work met the criteria for quality improvement and was exempt from institutional review board review.

## RESULTS

Seventy participants enrolled in the collaborative (36 physicians, 25 nurses/nurse practitioners, and 9 quality improvement consultants). Fifty-two (74%) participants attended at least three learning sessions. 56% of participants completed the presurvey, and 37% completed both the postsurvey and 1-year postcollaborative survey. For our primary outcome measure, 100% (15 of 15) of clinical divisions initiated local safety projects due to participating in the collaborative. We identified three major project themes: preventable harm at the bedside, processes of care, and communication and follow-up. Examples of local improvements that resulted from the collaborative-initiated safety projects included decreased rates of ICU transfer within 48 hours of direct floor admission from 1 in 36 to 1 in 60 patients and an increase in the percentage of oral chemotherapy prescriptions adherent to best practice guidelines on the Neuro-Oncology service from 64% to 72%. Seven physicians enrolled for Maintenance of Certification Part 4 credit.

For one secondary outcome measure, the percentage of survey respondents who reported feeling “completely confident” in their ability to improve safety increased from 26% (n = 39) to 58% (n = 26) from September 2020 to September 2021 (*P* = 0.01). The percentage of respondents who reported feeling “completely confident” in their ability to improve safety increased from 26% (N = 39) to 65% (N = 26) (*P* = 0.01) from precollaborative September 2020 to 1-year postcollaborative September 2022. We collected qualitative participant feedback and examples, including (1) “benefited from learning from others and hearing other peoples’ perspectives, successes and challenges,” (2) “the collaborative gave structure and tools to implement at the local level that has led to more robust safety management locally,” (3) “The collaborative helped increase communication inter-divisionally and identifying themes across the department,” and (4) “The collaborative supported multidisciplinary participation in safety work.”

**Fig. 4.**
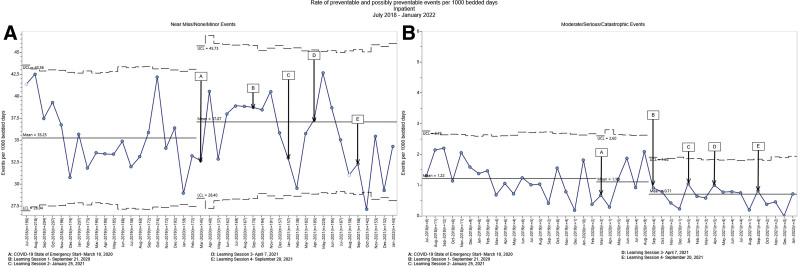
Statistical process control charts (u-charts) for inpatient preventable and possibly preventable event rates per 1000 bedded days. A, the rate of inpatient near miss/none/minor preventable and possibly preventable events per 1000 bedded days did not change throughout the intervention. B, the rate of inpatient moderate/serious/catastrophic events preventable and possibly preventable events per 1000 bedded days decreased from a mean rate of 1.10 (baseline period) to 0.71 (throughout the intervention period). UCL, upper control limit; LCL, lower control limit.

**Fig. 5.**
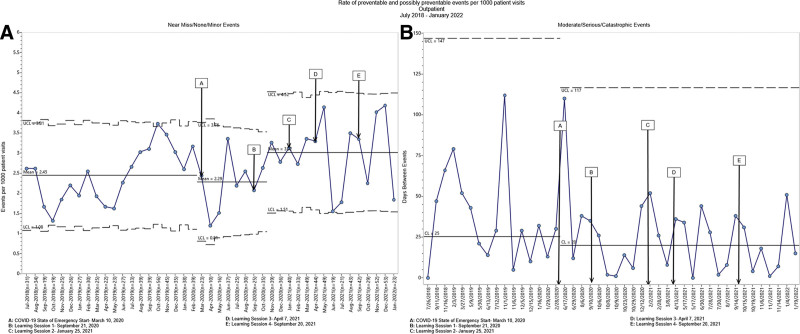
Statistical process control charts for outpatient preventable and possibly preventable events. A, the u-chart displays the rate of outpatient near miss/none/minor preventable and possibly preventable events per 1000 patient visits, which increased from a mean rate of 2.29–3.02 throughout the intervention period. B, the g-chart displays the median for days between events for outpatient moderate/serious/catastrophic events, which was 20 throughout the intervention period.

**Fig. 6.**
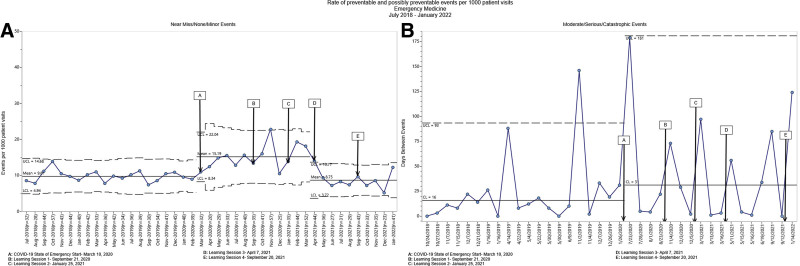
Statistical process control charts for preventable and possibly preventable emergency medicine events. A, the rate of emergency medicine near miss/none/minor preventable and possibly preventable events per 1000 patient visits decreased from a mean of 9.81 during the baseline period to 8.75 toward the end of the learning sessions and postcollaborative. However, there was a slight increase during the intervention period from March 2020 to March 2021 (mean = 15.19). B, the g-chart displays the median for days between events for emergency medicine moderate/serious/catastrophic events was 31 throughout the intervention period.

For another secondary outcome measure, we looked at safety event reporting rates across inpatient, outpatient and emergency settings. We observed decreased reported moderate/serious/catastrophic events in the inpatient setting and increased reported near miss/none/minor events in the outpatient and emergency department settings. However, we observed no other statistically significant changes in reporting, including near miss/none/minor preventable and possibly preventable events (Figs. 4–6).

## DISCUSSION

Using the Institute for Healthcare Improvement’s BTS model, we developed a structured, multidisciplinary learning collaborative to train frontline clinicians to manage safety. Engagement was strong over 4 learning sessions, leading to 15 important initiatives to reduce harm. We significantly increased participant-reported confidence in their ability to improve safety in their local areas. We observed decreased reports of high-level safety events and increased low-level reporting in some areas.

We selected the collaborative educational approach because similar efforts demonstrated potential for fostering learning and driving improvement in healthcare settings. Tuma et al launched an initiative to prevent healthcare-associated infections in intensive care units. They found that implementing BTS significantly reduced the incidence of central line-associated bloodstream infections, ventilation-associated pneumonia, and catheter-associated urinary tract infections.^11^ Desai et al found that BTS collaborative participation was associated with improved discharge instruction quality for hospitals with high baseline performance.^12^ These studies suggest that the BTS methodology is an important model for complex problem-solving in healthcare settings. Our application of the BTS model was novel in that we focused on training to boost frontline provider facility and confidence in safety rather than on a single clinical problem. Direct clinical outcomes are more challenging to measure using our approach. Still, we launched over a dozen safety projects and improved provider confidence in their ability to improve safety in their area. Of note, we observed decreased department-level moderate/serious/catastrophic safety data in the inpatient setting and an increase in the emergency department and outpatient near-miss/none/minor safety data. We cannot claim causation as we could not control all safety-related efforts at our institution that might have impacted the data. Also, event reporting is voluntary at our hospital, likely leading to underreporting, and the collaboration may have contributed to the increase in reporting in emergency and outpatient settings. A Global Trigger Tool (GTT) has been well-established for identifying adverse events in pediatric populations. In one previous study, a GTT demonstrated the ability to detect a rate of harm 2–3 times higher than published pediatric rates.^13^ Our institution is currently investigating GTT usage. Although reliance on reported data can be challenging, we helped broaden the scope of data capture by highlighting sources perhaps not previously considered. These sources include information collected in safety rounds, peer feedback, safety audits, simple safety conversations with staff, analysis of trends in performance measures and patient/parent feedback.

The most important aspect of this work was developing and applying the Safety Management Framework. Given the myriad sources of potential harm, a systematic approach to capturing, analyzing, and mitigating threats and events can be useful to local safety leaders. Provision of a practical framework that highlights the steps of input, throughput and output likely contributed to an increased sense of confidence in event and threat detection. It provided a path to actionable improvement efforts. Several participating divisions began implementing the framework as a new safety system evaluation method. For example, we captured the following comment from a physician, “Learning to apply a formal structure to safety has improved our ability to identify and address risks.”

This new approach requires a significant departure from traditional thinking, so many are slowly integrating the framework into their safety management workflows. Goldman et al introduced an alternative safety framework called The Measurement and Monitoring of Safety Framework, which aimed to move beyond a narrow focus on measurement and prior harmful events. This framework was developed and implemented via a learning collaborative on a much broader scale (across multiple organizations instead of across divisions within one organization). They experienced similar limited evidence of impact due to the dramatic departure from traditional safety strategies focusing on discrete problems.^14^ This speaks to the need for continued monitoring and frequent coaching on using novel frameworks that take a nontraditional approach to safety management.

The most exciting outcome of our work was initiating 15 divisional safety projects. Instituting a project component allowed participants to apply lessons from the learning sessions although potentially increasing safety in their areas. In the year before our collaboration, only 10 safety projects were underway, and most divisions were not actively working on safety projects. As a result of our intervention, all divisions simultaneously had a safety project in progress.

We found success in respecting divisional culture and capabilities by considering the Model for Understanding Success in Quality and its emphasis on context.^15^ Local safety leaders determined where to focus their harm reduction efforts. They had the opportunity to obtain feedback on their efforts through regular report-outs and check-ins with colleagues in the learning sessions. They received additional motivation to keep their efforts moving forward. This helped establish a nascent safety community within the department, which will continue to foster sharing and learning.

We utilized a virtual platform effectively during the COVID-19 pandemic by implementing several best practices. These included establishing ground rules and expectations, leveraging platform tools such as breakout rooms for small group discussions, regularly pausing to enhance virtual interactions, and conducting postsession surveys for participant feedback.^16^

Although we preferred in-person collaboration, previous studies demonstrated the value and uptake of virtual safety education in healthcare settings. Ortega et al found that implementing an international online Nursing and Patient Safety Course was well-received by participants and resulted in increased knowledge of patient safety. This highlighted the potential of online distance learning to enhance patient safety education for clinicians.^17^ Virtual learning collaboratives have also been shown to boost healthcare providers’ confidence in their abilities to carry out tasks related to improvement work. Oliver et al demonstrated that participants gained confidence in HPV vaccine communication and quality improvement skills.^18^ Scott et al also demonstrated that a facilitated, virtual quality improvement learning collaborative was an effective way to improve safe sleep counseling among a diverse group of pediatric practices.^19^

## LIMITATIONS

Our study has several limitations. The safety learning collaborative was carried out in a single pediatric hospital department of pediatrics; therefore, the results and experience may not be generalizable to other environments. Although many of the new safety projects led to improved safety, we have not yet been able to measure the outcomes of all projects because many are still underway. In addition, the survey response rates could have been better, likely due to the anonymous survey approach, virtual format, and the COVID-19 pandemic. Based on the anonymity of the survey, which was important, we could not directly compare individual scores. We were, therefore, unable to identify who did not respond to our survey, which prevented performing any mix adjustments for the results. In addition, we were unable to control for other safety interventions. Therefore, unmeasured safety efforts may have improved participant confidence and inpatient safety. Although we were pleased to observe a reduction in high-level harm in the inpatient setting, we could not fully attribute this to our efforts alone. Whether this work will ultimately lead to safer care is still being determined. However, by building confidence in frontline providers’ safety, we hope they will have moved further along the road to high reliability.

## CONCLUSIONS

We developed and implemented a virtual learning collaborative to improve patient safety in a children’s hospital pediatric department. Our work led directly to the initiation of many safety projects. Coupling focused learning activities with safety improvement projects can reinforce the understanding and application of fundamental safety concepts among frontline safety leadership. We also improved participants’ confidence in safety management and noted a decrease in serious inpatient events. This effort may be foundational in improving safety in a children’s hospital department of pediatrics.

## ACKNOWLEDGMENTS

We thank Donald M. Berwick, MD, MPP, FRCP, KBE, and Gary R. Fleisher, MD, for their participation in the collaboration and their call to reduce harm. We want to thank our data analyst, Ben Ethier, for his contributions. Finally, we would like to express our appreciation for the commitment to safe care and quality improvement demonstrated by the physicians, nurses, and quality improvement consultants who participated in the safety learning collaborative.

## Supplementary Material


