# Supplementary figures and images for: Improving Safety through a Virtual Learning Collaborative
Source: Pediatr Qual Saf. 2024 Jul 19;9(4):e740. doi: 10.1097/pq9.0000000000000740 (PMC11259400; doi:10.1097/pq9.0000000000000740)

# Department of Pediatrics Quality Improvement Work Structure

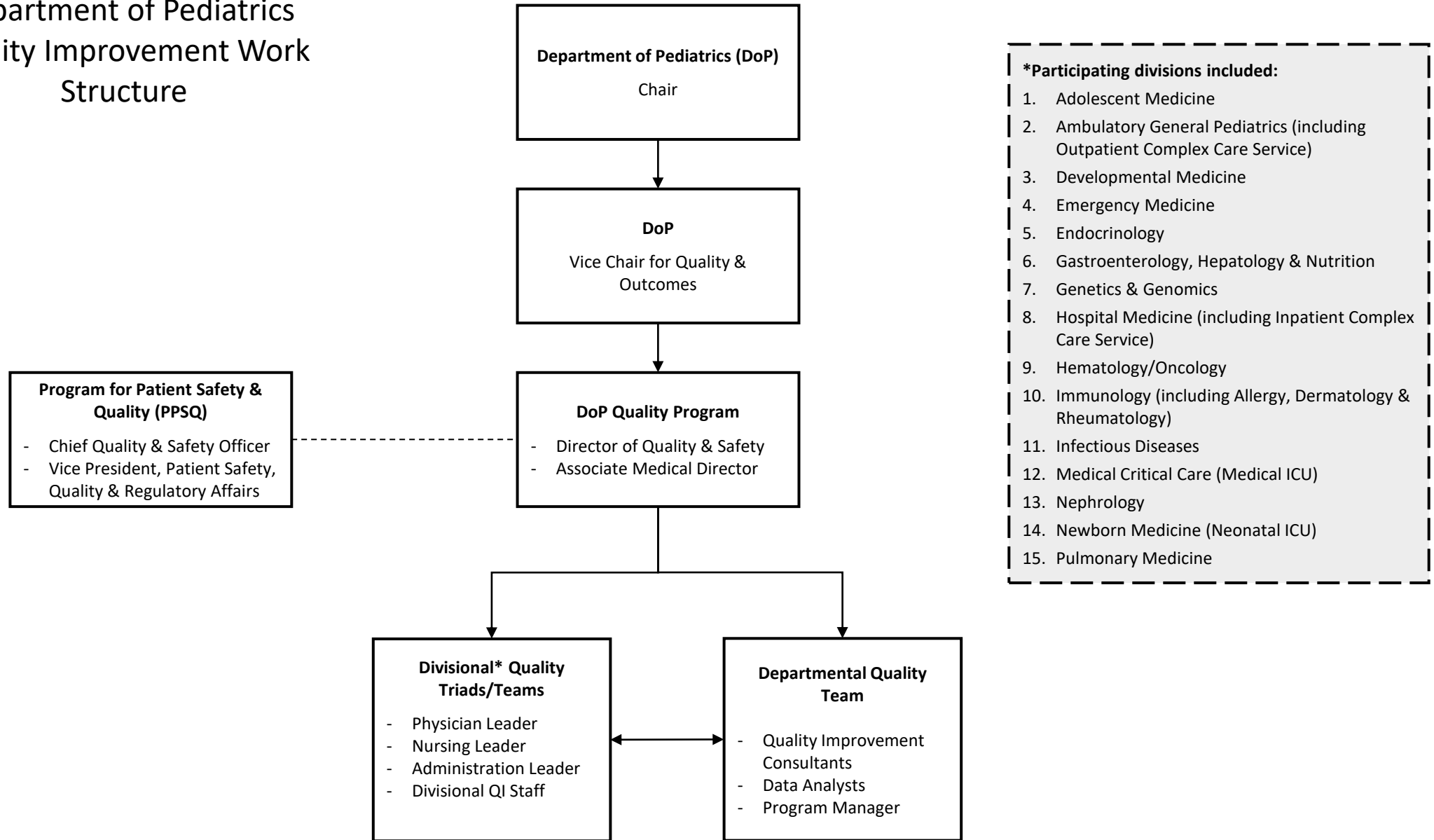

Supplement: Supplementary file 1 [file pqs-9-e740-s001.pdf]
