# Supplementary material for: Improving Safety through a Virtual Learning Collaborative
Source: Pediatr Qual Saf. 2024 Jul 19;9(4):e740. doi: 10.1097/pq9.0000000000000740 (PMC11259400; doi:10.1097/pq9.0000000000000740)
Supplement: Supplementary file 2 [file pqs-9-e740-s002.pdf]

Event Classification Levels

Severity	Level 0 Near Miss	Intercepted before reaching the patient
	Level 1 None	No change in condition, no intervention indicated
	Level 2 Minor	Transient change in condition, not life threatening, condition returns to baseline, required monitoring, required minor intervention
	Level 3 Moderate	Transient change in condition, may be life threatening if not treated, condition returns to baseline, required monitoring, required intervention
	Level 4 Major	Change in condition, life threatening if not treated, change in condition may be permanent, may have required initial or readmit to hospital, may have required transfer to ICU, required monitoring, required major intervention
	Level 5 Catastrophic	Death
Preventability	Not Preventable	Events where no obvious breach of standard professional behavior or technique occurred, necessary precautions were taken, no clearly known alteration in method or care exists to prevent the event
	Possibly Preventable	Events where definite breach of standard professional behavior or technique was not identified but may have occurred, necessary precautions may not have been taken, event may have been preventable by modification of behavior technique or care
	Preventable	Events where definite breach of standard professional behavior or technique was identified, necessary precautions were not taken, event was preventable by modification of behavior technique or care
