# Supplementary material for: Improving Safety through a Virtual Learning Collaborative
Source: Pediatr Qual Saf. 2024 Jul 19;9(4):e740. doi: 10.1097/pq9.0000000000000740 (PMC11259400; doi:10.1097/pq9.0000000000000740)
Supplement: Supplementary file 3 [file pqs-9-e740-s003.pdf]

Pre and Post Collaborative Survey Questions: Checkmark indicates inclusion of question in the survey.

Question	Answer Scale	Pre-Collaborative	Post-Collaborative	Post-Collaborative (1 year)
Select your role.	• Physician<br>• Nursing (RN,NP)<br>• Quality Improvement Consultant (QIC)<br>• Other (write in)	✓	✓	✓
Select your division.	• Adolescent Medicine<br>• Ambulatory General Pediatrics (including Outpatient Complex Care Service)<br>• Developmental Medicine<br>• Emergency Medicine<br>• Endocrinology<br>• Gastroenterology, Hepatology & Nutrition<br>• Genetics & Genomics<br>• Hospital Medicine (including Inpatient Complex Care Service)<br>• Hematology/Oncology<br>• Immunology (including Allergy, Dermatology & Rheumatology)<br>• Infectious Diseases<br>• Medical Critical Care (Medical ICU)<br>• Nephrology<br>• Newborn Medicine (Neonatal ICU)<br>• Pulmonary Medicine	✓	✓	✓
I feel confident in my ability to use the hospital's safety reporting system.	• Completely confident<br>• Fairly confident<br>• Somewhat confident<br>• Slightly confident<br>• Not confident at all<br>• I do not use the hospital's safety reporting system.	✓	✓	✓
My division develops improvement initiatives from themes identified from low level (0-2) event review.	• Always<br>• Very Often<br>• Sometimes<br>• Rarely<br>• Never<br>• Unsure	✓	✓	✓
I feel confident in my ability to use the "Safety Framework" to help my safety team's effectiveness in my area.	• Completely confident<br>• Fairly confident<br>• Somewhat confident<br>• Slightly confident<br>• Not confident at all<br>• I do not use the hospital's safety reporting system		✓	✓

Question	Answer Scale	Pre-Collaborative	Post-Collaborative	Post-Collaborative (1 year)
I feel confident using high reliability tools (e.g., apparent cause analysis, fishbone diagrams etc.) to analyze safety events.	• Completely confident<br>• Fairly confident<br>• Somewhat confident<br>• Slightly confident<br>• Not confident at all<br>• In my role, I do not analyze safety events	✓	✓	✓
I feel confident that our team has robust mechanisms, in addition to events, to identify cases for review.	• Completely confident<br>• Fairly confident<br>• Somewhat confident<br>• Slightly confident<br>• Not confident at all<br>• In my role, I do not participate in identifying cases for review	✓	✓	✓
I feel confident leading/actively participating in Morbidity and Mortality (M&M) conferences.	• Completely confident<br>• Fairly confident<br>• Somewhat confident<br>• Slightly confident<br>• Not confident at all<br>• In my role, I do not participate in M&M conferences	✓	✓	✓
M&M conferences in my division follow a standardized process.	• Always<br>• Very Often<br>• Sometimes<br>• Rarely<br>• Never<br>• In my role, I do not participate in M&M conferences	✓	✓	✓
My division has a fair and just safety culture.	• Always<br>• Very Often<br>• Sometimes<br>• Rarely<br>• Never<br>• Unsure	✓	✓	✓
I feel confident in my ability to improve safety within my division.	• Completely confident<br>• Fairly confident<br>• Somewhat confident<br>• Slightly confident<br>• Not confident at all	✓	✓	✓
As a result of participation in this collaborative, harm has been reduced in my area.	• Strongly agree<br>• Somewhat agree<br>• Neutral<br>• Somewhat disagree<br>• Strongly disagree		✓	✓

Question	Answer Scale	Pre-Collaborative	Post-Collaborative	Post-Collaborative (1 year)
One year after the safety collaborative, I feel participation improved safety management in my area.	• Strongly agree<br>• Somewhat agree<br>• Neutral<br>• Somewhat disagree<br>• Strongly disagree			✓
Are there any other comments you would like to share regarding safety management within your division?	Open-ended response	✓	✓	
Are there any comments/feedback you would like to share with us about participating in this collaborative?	Open ended response		✓	
Please add any questions or comments below.	Open ended response			✓
